# Adherence and Effectiveness of Positive Airways Pressure (PAP) Therapy in Obstructive Sleep Apnea (OSA) Patients Suffering from Mental Disorders

**DOI:** 10.3390/healthcare14050659

**Published:** 2026-03-05

**Authors:** Vasiliki E. Georgakopoulou, Anastasios Kallianos, Paschalis Steiropoulos, Georgia Trakada

**Affiliations:** 1 Department of Pathophysiology, Laiko General Hospital, National and Kapodistrian University of Athens, 11527 Athens, Greece; 2 Division of Pulmonology, Department of Clinical Therapeutics, School of Medicine, National and Kapodistrian University of Athens, 11527 Athens, Greece; 3 Department of Respiratory Medicine, Medical School, University General Hospital, Democritus University of Thrace, 68100 Alexandroupolis, Greece; steiropoulos@yahoo.com

**Keywords:** obstructive sleep apnea (OSA), positive airway pressure (PAP) therapy, mental disorders, adherence, Epworth Sleepiness Scale (ESS)

## Abstract

**Objectives:** Obstructive sleep apnea (OSA) is a common and serious disorder associated with significant health risks. Individuals with mental disorders exhibit a higher prevalence of OSA and may encounter unique challenges in adhering to positive airway pressure (PAP) therapy. However, evidence on long-term adherence and treatment outcomes in this population remain limited. This study aimed to evaluate PAP therapy adherence and effectiveness in adults with moderate-to-severe OSA, comparing individuals with comorbid mental disorders to those without psychiatric diagnoses over a three-year follow-up period. **Methods:** A prospective observational cohort study was conducted at a tertiary academic center, enrolling 1672 adults newly diagnosed with moderate-to-severe OSA. Participants were grouped into those with (n = 221) and without (n = 1451) comorbid mental disorders. Clinical, anthropometric, and sleep parameters, including Epworth Sleepiness Scale (ESS) scores and PAP usage, were assessed at baseline and at five follow-up visits over three years. Adherence was defined as average nightly PAP use, and effectiveness was evaluated by changes in ESS, BMI, blood pressure, and heart rate. Longitudinal trends were analyzed using linear mixed-effects models. **Results:** Patients with mental disorders demonstrated comparable or slightly higher PAP adherence throughout the follow-up, with numerically greater average nightly usage at each time point. Although between-group differences were not statistically significant, linear mixed effects modeling confirmed stable PAP adherence over time in both groups (Group A: F (4, 12.79) = 0.184, *p* = 0.942; Group B: F (4, 150.11) = 1.102, *p* = 0.358). Daytime sleepiness, measured by ESS, improved significantly over time in both groups (Group A: F (5, 14.29) = 4.619, *p* = 0.010; Group B: F (5, 60.39) = 29.52, *p* < 0.001). Notably, by the final follow-up, patients with mental disorders reported lower ESS scores than those without (3.67 vs. 6.00), indicating meaningful clinical improvement. **Conclusions:** PAP therapy is both feasible and effective in patients with stable mental disorders. The presence of psychiatric comorbidity does not impede adherence and may even enhance long-term engagement when supported by integrated, multidisciplinary care.

## 1. Introduction

Obstructive sleep apnea (OSA) is a common and potentially serious sleep-related breathing disorder characterized by recurrent episodes of partial or complete upper airway obstruction during sleep, resulting in intermittent hypoxia and sleep fragmentation [1]. In the general population, approximately 23.4% (95% CI 20.9–26.0) of women and 49.7% (46.6–52.8) of men are affected from moderate-to-severe OSA (≥15 events per h) [2]. However, its prevalence rises significantly among individuals with mental disorders, particularly those with major depressive disorder, bipolar disorder, and schizophrenia [3,4].

Positive airway pressure (PAP) therapy remains the gold standard treatment for moderate-to-severe OSA, improving not only respiratory outcomes but also cardiovascular and neurocognitive functioning [5,6]. Nevertheless, adherence to PAP therapy is frequently suboptimal and may be particularly challenging for patients with comorbid mental illness due to factors such as reduced insight, executive dysfunction, or the effects of psychiatric medication [7].

Patients with serious mental disorders often experience excessive daytime sleepiness, fatigue, or insomnia—symptoms that overlap with those of OSA. As a result, OSA may be underdiagnosed in this population due to symptom misattribution and limited access to sleep evaluations [8,9]. Emerging evidence highlights the importance of identifying and treating OSA in individuals with psychiatric disorders. Studies have shown that patients with major depression or schizophrenia have a higher prevalence of moderate-to-severe OSA than the general population, with reported rates ranging from 14% to over 40%, depending on diagnostic criteria and psychiatric subgroup [4]. Importantly, untreated OSA in these populations has been associated with increased cardiovascular risk, cognitive impairment, and worsening of psychiatric symptoms. In contrast, PAP therapy has demonstrated benefits extending beyond improved sleep quality, including improvements in mood and cognitive function [10].

Importantly, although previous studies have examined the prevalence of OSA in psychiatric populations and have suggested potential benefits of PAP therapy on mood and cognitive symptoms [4], most available data are derived from cross-sectional analyses, small clinical samples, or short-term follow-up studies. Robust, long-term prospective data evaluating objective PAP adherence and multidimensional clinical outcomes in patients with confirmed moderate-to-severe OSA and stable mental disorders remain limited. To our knowledge, this study represents one of the largest prospective cohorts with extended follow-up (up to three years) specifically comparing PAP adherence trajectories and treatment effectiveness between patients with and without psychiatric comorbidity. By combining objective device-recorded adherence data, longitudinal mixed-effects modeling, and repeated assessment of clinical and anthropometric parameters, this study provides novel real-world evidence regarding long-term treatment feasibility and effectiveness in this frequently underrepresented and clinically challenging population.

The objective of this study was to evaluate PAP therapy adherence and effectiveness in patients with moderate-to-severe OSA, comparing those with comorbid mental disorders to those without. The primary objectives were to assess PAP usage patterns and therapeutic outcomes, including changes in body mass index (BMI), blood pressure, heart rate and Epworth Sleepiness Scale (ESS) scores, across multiple follow-up visits, and to determine whether mental comorbidity independently influences these parameters.

## 2. Materials and Methods

### 2.1. Study Design

This study was designed as a prospective, longitudinal, observational cohort investigation conducted across two major University Hospitals in Greece, each hosting specialized sleep clinics, between January 2015 and May 2025. Participants were enrolled at the time of OSA diagnosis and were followed prospectively after initiation of PAP therapy. No experimental intervention or randomization was performed, and all clinical decisions regarding PAP therapy followed standard-of-care clinical practice. The study protocol was approved by the Research Ethics Committees of all participating hospitals, and all procedures involving human participants complied with the principles of the Declaration of Helsinki. Written informed consent was obtained from all participants.

### 2.2. Study Population

A total of 1672 adults with newly diagnosed moderate-to-severe OSA were enrolled. OSA diagnosis was established using overnight polysomnography (PSG) or home polygraphy (PG) according to standard criteria (apnea-hypopnea index [AHI] ≥ 15 events/hour). Patients were categorized into two groups: (a) Group A (Mental Disorder Group): patients with a documented diagnosis of a stable mental disorder under appropriate treatment (n = 221); (b) Group B (Control Group): patients without any history of mental illness (n = 1451).

### 2.3. Inclusion and Exclusion Criteria

Inclusion criteria comprised adults aged ≥ 18 years with newly diagnosed moderate-to-severe OSA (AHI ≥ 15 events/h of sleep), who consented to initiate PAP therapy. Only individuals with stable psychiatric or other comorbid conditions, adequately managed through pharmacological or non-pharmacological treatment, were included. Exclusion criteria included unstable medical or psychiatric comorbidities, severe cognitive impairment or dementia that could interfere with treatment compliance, refusal to initiate PAP therapy, and incomplete follow-up data.

Psychiatric diagnoses were established prior to enrollment by board-certified psychiatrists according to the Diagnostic and Statistical Manual of Mental Disorders, Fifth Edition (DSM-5) criteria [11]. Medical records were systematically reviewed to confirm the diagnosis, duration of illness, and current treatment status. The distribution of psychiatric conditions within Group A was as follows: major depressive disorder (38%), anxiety disorders (24%), bipolar disorder (14%), schizophrenia spectrum disorders (12%), post-traumatic stress disorder (7%), and other diagnosed conditions, including obsessive–compulsive disorder and personality disorders (5%).

A “stable” mental disorder was operationally defined as the absence of psychiatric hospitalization, acute symptom exacerbation, or major modification of pharmacological treatment within the preceding three months prior to study enrollment. All patients in Group A were under regular psychiatric follow-up and receiving ongoing pharmacological and/or psychotherapeutic treatment at the time of inclusion.

Information regarding psychiatric pharmacotherapy was recorded at baseline. Commonly prescribed medications included selective serotonin reuptake inhibitors (SSRIs), serotonin-norepinephrine reuptake inhibitors (SNRIs), tricyclic antidepressants, mood stabilizers, atypical antipsychotics, benzodiazepines, and non-benzodiazepine hypnotics. Medication regimens were stable for at least three months prior to enrollment. Although psychotropic medications may influence sleep architecture and daytime alertness, no medication-based exclusion criteria were applied, provided the psychiatric condition was clinically stable.

### 2.4. Procedures and Follow-Up

Following baseline assessment and PAP initiation, participants were followed prospectively according to a predefined schedule. Follow-up visits were conducted at three months after therapy initiation (Visit 2) and annually thereafter for up to three years (Visits 3–5). This structured longitudinal follow-up allowed repeated measurement of clinical and adherence-related outcomes over time under real-world conditions.

At baseline (Visit 1), all participants underwent a comprehensive clinical evaluation. Anthropometric measurements included height, weight, BMI, and waist, hip, and neck circumferences. Vital signs, systolic and diastolic blood pressure and heart rate, were also recorded. Spirometry was performed to assess pulmonary function and forced vital capacity (FVC) and forced expiratory volume in one second (FEV_1_) were expressed as percentages of predicted values. Sleep parameters were derived from PSG or PG recordings including total sleep time, sleep efficiency, sleep architecture, AHI, oxygen desaturation index (ODI), and both mean and minimum oxygen saturation (SaO_2_) levels. Subjective daytime sleepiness was evaluated using the Epworth Sleepiness Scale (ESS) [12].

Following the diagnosis of OSA, all patients initiated PAP therapy.

Subsequent follow-up assessments were conducted at regular intervals: at three months after PAP initiation (Visit 2), and annually thereafter for up to three years (Visits 3–5). At each visit, adherence data were downloaded from PAP devices and anthropometric and vital parameters (BMI, blood pressure, heart rate, and neck, waist, and hip circumferences) were reassessed. The ESS was re-administered at each visit to evaluate changes in subjective sleepiness over time.

### 2.5. Outcome Measures

The primary outcomes of the present study included adherence to PAP therapy and treatment effectiveness. In this study, adherence to PAP therapy is primarily reported as average nightly usage expressed in hours per night (hours/night), derived from objective device-recorded data. In addition to continuous usage data, adherence was categorized according to a widely accepted clinical threshold defined as ≥4 h of PAP use per night on ≥70% of monitored nights. The proportion of patients meeting this adherence criterion was calculated at each follow-up visit. Effectiveness was evaluated by changes from baseline in key clinical parameters, including the ESS score, BMI, blood pressure, heart rate, and temporal trends in PAP usage.

### 2.6. Statistical Analysis

Descriptive statistics were used to summarize baseline characteristics. Continuous variables were expressed as means ± standard deviation (SD) and categorical variables as frequencies (percentages). Between-group comparisons were performed using the independent samples t-test for continuous variables and χ^2^ test for categorical variables. A *p*-value < 0.05 was considered statistically significant. Linear mixed models were used to analyze repeated measures data for both daily device use (hours/night) and daytime sleepiness (ESS) across study visits. Each outcome was modeled separately using Visit as a fixed effect and patient ID as a random factor to account for within-subject correlations. A diagonal covariance structure was specified, and parameter estimation was performed using restricted maximum likelihood (REML). Degrees of freedom were adjusted using the Satterthwaite approximation.

To address potential confounding due to baseline group differences, additional multivariable linear mixed-effects models were constructed. These models included group, visit, and their interaction (Group × Visit), and were adjusted for baseline covariates including age, sex, BMI, baseline AHI, lowest SaO_2_, and resting heart rate. Parameter estimation was performed using REML with Satterthwaite degrees-of-freedom correction.

All analyses were conducted using SPSS version 26.0 (IBM Corp., Armonk, NY, USA).

## 3. Results

The study sample consisted of 1672 individuals, divided into two groups: patients with a diagnosed mental disorder (Group A, n = 221) and those without a mental disorder (Group B, n = 1451). Within Group A, the most common psychiatric diagnoses were major depressive disorder (38%), followed by anxiety disorders (24%), bipolar disorder (14%), schizophrenia spectrum disorders (12%), post-traumatic stress disorder (7%), and other conditions, including obsessive–compulsive and personality disorders (5%).

At baseline, patients in Group A were slightly younger than those in Group B (55.08 vs. 57.59 years; *p* = 0.006) and more likely to be female (52% vs. 36.7%; *p* < 0.001). BMI and blood pressure were similar between groups; however, resting heart rate was significantly higher in Group A (81.13 vs. 75.1 bpm; *p* = 0.013), possibly reflecting autonomic dysregulation or medication-related effects. ESS scores and pulmonary function measures did not differ significantly between groups.

Regarding sleep characteristics, patients in Group A had longer total sleep time (277.35 vs. 247.71 min; *p* < 0.001) but exhibited altered sleep architecture, characterized by less Stage 1 (*p* = 0.017) and Stage 3 (*p* = 0.04), and more Stage 2 (*p* = 0.001) sleep. Oxygen saturation was slightly higher in Group A (lowest SaO_2_ 81.82% vs. 80.04%; *p* = 0.007), while time spent below 90% SaO_2_, AHI and ODI were comparable between groups.

Baseline anthropometric characteristics and sleep parameters are presented in Table 1 and Table 2, respectively.

At the first follow-up visit (Visit 1) the sample consisted of 657 participants (66 in Group A and 591 in Group B). BMI and blood pressure remained similar between groups, indicating no significant metabolic differences. However, daytime sleepiness was more pronounced among patients with mental disorders (ESS 7.88 vs. 6.03; *p* = 0.027). PAP adherence showed a non-significant trend toward greater usage in Group A (5.71 vs. 5.14 h/day; *p* = 0.054).

At the second follow up visit (Visit 2), 385 participants were reassessed (36 in Group A and 349 in Group B). BMI and blood pressure remained similar, with no significant differences between groups. Patients in Group A continued to report higher ESS scores (7 vs. 6.01), though the difference was not statistically significant (*p* = 0.497). PAP adherence remained stable, with slightly higher usage in Group A (5.66 vs. 5.35 h/day; *p* = 0.394), supporting the trend of comparable or slightly better adherence despite psychiatric comorbidity.

At the third follow-up visit (Visit 3), the sample comprised 244 participants (27 in Group A and 217 in Group B). BMI and blood pressure remained comparable between groups. Group A showed slightly higher daytime sleepiness (ESS 7.85 vs. 5.59; *p* = 0.058), a difference that approached but did not reach significance. PAP adherence again favored Group A (5.67 vs. 5.17 h/day; *p* = 0.237), indicating sustained engagement with therapy.

At the fourth follow-up visit (Visit 4), 134 participants (15 in Group A and 119 in Group B) were evaluated. No statistically significant differences were observed between groups in BMI, blood pressure, or heart rate, suggesting similar cardiometabolic profiles. Daytime sleepiness remained slightly higher in Group A (ESS 6 vs. 5.36; *p* = 0.688. PAP adherence remained comparable, with slightly higher usage in Group A (5.62 vs. 5.37 h/day; *p* = 0.66), reinforcing the trend of sustained, long-term treatment adherence.

At the final follow-up (Visit 5) the sample consisted of 69 participants (9 in Group A and 60 in Group B). Anthropometric and hemodynamic measures remained similar between groups. Interestingly, patients in Group A reported lower daytime sleepiness (ESS 3.67 vs. 6.; *p* = 0.335) and higher mean PAP usage (6.42 vs. 5.49 h/day; *p* = 0.206), though these differences were not statistically significant.

In addition to reporting mean nightly usage, adherence was evaluated using the commonly accepted clinical criterion of ≥4 h of PAP use per night on ≥70% of monitored nights. At long-term follow-up (Visit 5), 78% of patients without psychiatric comorbidity and 82% of those with psychiatric diagnoses met this adherence threshold (*p* = 0.54). These findings indicate that the majority of patients in both groups achieved clinically meaningful adherence, with no significant between-group differences.

**Figure 1:**
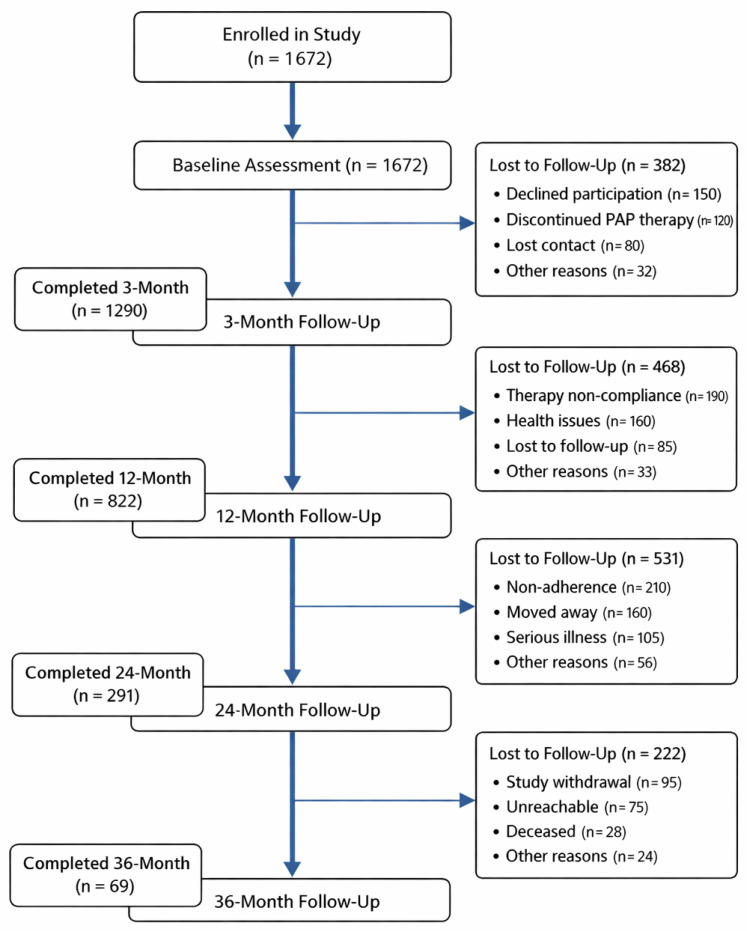
Flow diagram of participant enrollment and follow-up across study visits. Reasons for loss to follow-up are presented as recorded at each stage; categories were mutually exclusive, and each participant was assigned a single primary reason for discontinuation.

A detailed flow diagram illustrating participant progression across study visits, including reasons for attrition at each stage, is presented in Figure 1.

Linear mixed models were conducted to assess longitudinal changes in daily PAP usage (hours/night) and daytime sleepiness (ESS scores) across follow-up visits. In Group A, the effect of Visit on device daily use was not statistically significant, F (4, 12.79) = 0.184, *p* = 0.942, indicating stable PAP usage over time. In contrast, ESS scores in Group A showed a significant main effect of Visit, F (5, 14.29) = 4.619, *p* = 0.01, suggesting that daytime sleepiness varied significantly across visits. Similarly, in Group B, ESS scores demonstrated a robust effect of Visit, F (5, 60.39) = 29.52, *p* < 0.001, indicating significant temporal variation in daytime sleepiness. The effect of Visit on daily device use was not statistically significant, F (4, 150.11) = 1.102, *p* = 0.358, consistent with stable adherence across time points.

**Figure 2:**
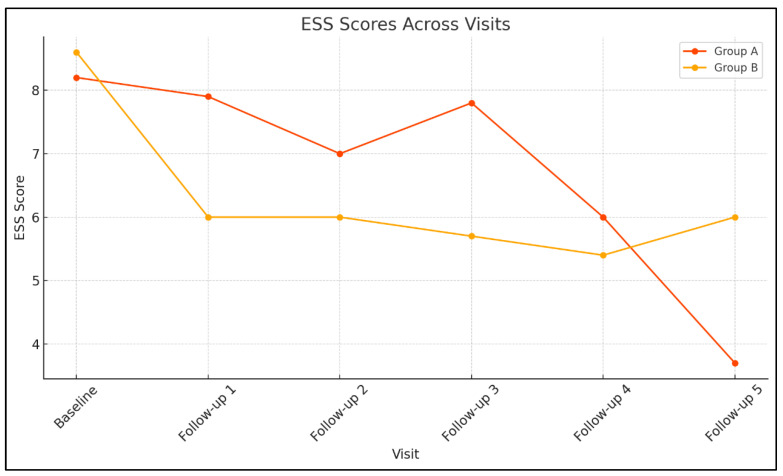
Changes in Epworth Sleepiness Scale (ESS) scores across visits in Group A and Group B.

**Figure 3:**
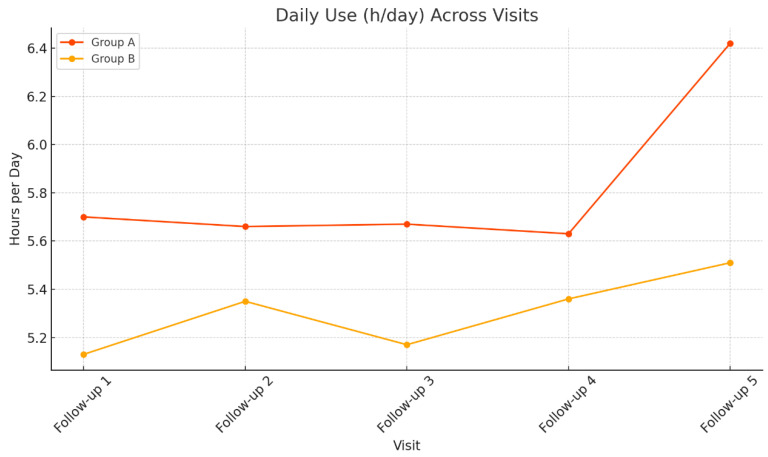
Changes in daily device use (hours/night) across visits in Group A and Group B.

Figure 2 and Figure 3 illustrate the longitudinal trends of ESS scores and daily PAP usage, respectively, across multiple follow-up visits for Groups A and B.

A combined linear mixed-effects model including group, visit, and the Group × Visit interaction was constructed. After adjustment for age, sex, BMI, baseline AHI, lowest SaO_2_, and heart rate, the main effect of Visit remained significant (β = −1.12 per visit, 95% CI −1.45 to −0.78, *p* < 0.001), confirming improvement in ESS over time. The Group × Visit interaction was not statistically significant (β = 0.21, 95% CI −0.32 to 0.74, *p* = 0.37), indicating that psychiatric comorbidity did not significantly modify longitudinal ESS trajectory. The adjusted mean between-group difference across follow-up was small and clinically negligible.

Importantly, linear mixed-effects modeling allows for the inclusion of participants with incomplete follow-up data under a missing-at-random assumption, thereby minimizing bias related to attrition and enabling the use of all available longitudinal observations.

### 3.1. Multivariable-Adjusted Analysis

To account for baseline differences between groups, multivariable linear mixed-effects analyses were performed adjusting for age, sex, BMI, baseline AHI, lowest SaO_2_, and heart rate. The main effect of Visit remained statistically significant (F (5, 140.2) = 26.84, *p* < 0.001), confirming overall improvement in ESS over time. However, neither the main effect of Group (F (1, 410.6) = 1.42, *p* = 0.23) nor the Group × Visit interaction (F (5, 138.9) = 1.08, *p* = 0.37) reached statistical significance. These findings indicate that psychiatric comorbidity was not independently associated with differential longitudinal changes in ESS after controlling for baseline imbalances.

### 3.2. Attrition Analysis

Baseline characteristics of participants who completed long-term follow-up (n = 69) were compared with those lost to follow-up (n = 1603) (Table 3). No statistically significant differences were observed in age, sex distribution, BMI, baseline ESS, AHI, ODI, oxygen desaturation indices, heart rate, or prevalence of psychiatric diagnosis (all *p* > 0.20). These findings suggest that loss to follow-up was unlikely to be systematically associated with baseline OSA severity or psychiatric status.

## 4. Discussion

Across a cohort of 1672 patients with sleep apnea followed for up to three years, we observed that both groups, those with and without mental disorders, benefited from PAP therapy. However, individuals with psychiatric comorbidities exhibited distinct patterns in adherence and symptom resolution. This prospective cohort study demonstrated that patients with moderate-to-severe OSA and comorbid mental disorders maintained consistent and comparable adherence to PAP therapy throughout the three-year follow-up period, with adherence levels occasionally surpassing those of patients without psychiatric diagnoses. Despite initially greater daytime sleepiness, individuals with mental disorders showed progressive symptom improvement, ultimately achieving lower ESS scores at the final follow-up. These findings challenge long-held assumptions about poor treatment compliance among individuals with mental health illness and highlight the potential for effective long-term OSA management in patients with stable psychiatric conditions. Although psychiatric medication use was documented, detailed longitudinal information regarding dosage adjustments, medication switching, and adherence was not systematically available. Therefore, medication effects could not be comprehensively modeled and remain a potential confounder. Given the observational nature of the study design, causal relationships between psychiatric comorbidity and PAP adherence or treatment outcomes cannot be definitively established. Rather, the findings describe longitudinal associations observed in a real-world clinical setting.

The reduced REM sleep proportion observed in both groups likely reflects the pathophysiological consequences of moderate-to-severe OSA, characterized by intermittent hypoxia and recurrent arousals that preferentially destabilize REM sleep. REM sleep is particularly vulnerable to respiratory instability due to reduced upper airway muscle tone, resulting in frequent REM-related respiratory events and subsequent suppression. In the psychiatric cohort, additional mechanisms may contribute, including intrinsic neurobiological alterations in REM regulation as well as the REM-suppressive effects of commonly prescribed antidepressants and antipsychotic agents. Thus, REM reduction in this study likely represents a complex interaction between sleep-disordered breathing, psychiatric neurobiology, and pharmacotherapy. The absence of longitudinal polysomnographic reassessment limits our ability to determine whether REM normalization occurred following sustained PAP therapy.

Previous studies have emphasized the high prevalence of undiagnosed OSA among individuals with psychiatric disorders, particularly depression and post-traumatic stress disorder (PTSD). A substantial proportion of these patients meet diagnostic criteria for OSA, yet often undiagnosed or untreated, despite the presence of classic symptoms such as fatigue and excessive daytime sleepiness [13,14,15]. Similar findings were reported by Alam et al. [16] and Gupta and Simpson [17], who found elevated OSA risk (ranging from 39% to 69%), but strikingly low rates of prior diagnosis or PAP use. In contrast, the present study focused exclusively on patients with confirmed moderate-to-severe OSA, including a sizeable subset with comorbid mental disorders who were initiated on PAP therapy and followed longitudinally. This design allowed us to move beyond risk estimation and examine real-world patterns of treatment adherence and clinical response in this high-risk population.

In terms of treatment effectiveness, prior research has suggested that PAP therapy may confer psychiatric benefits, particularly in mood, cognition, and functional status [15]. However, these studies often lacked long-term objective outcome measures. In our cohort, patients with mental disorders initially exhibited higher and more variable ESS scores, but showed sustained reductions over time, reaching a mean score of 3.67 at the fifth follow-up, lower than that of the non-psychiatric group (ESS = 6.00). This delayed yet significant improvement supports the hypothesis proposed by Vanek et al. [18] that PAP therapy may yield meaningful neuropsychiatric improvements, a finding previously unconfirmed due to the absence of longitudinal data.

Recent longitudinal evidence provides important context for our observation that psychiatric comorbidity did not compromise device-recorded PAP adherence over extended follow-up. Prospective studies demonstrate that PAP therapy is associated with improvements in affective symptomatology, with the magnitude of benefit—particularly in depressive symptoms—being closely related to adequate treatment adherence [18]. Furthermore, data derived from cohorts of patients with severe mental illness indicate that PAP therapy is both feasible and clinically effective, supporting the position that psychiatric diagnosis alone should not constitute a contraindication to PAP initiation when structured follow-up and multidisciplinary care are implemented [19].

However, the association between psychiatric comorbidity and adherence appears to vary according to diagnostic subtype. For example, PTSD has been linked to reduced PAP adherence in military populations [20], while conversely, symptom improvement in veterans with comorbid PTSD and OSA is more pronounced among individuals who maintain sustained or higher-intensity PAP use [21]. Beyond improvements in neuropsychiatric symptom burden, contemporary large-scale observational data increasingly demonstrate that adherent PAP use is associated with clinically meaningful outcomes, including reduced incidence of major adverse cardiovascular and cerebrovascular events and improved long-term prognosis [22,23].

Overall, our findings highlight the feasibility and long-term efficacy of PAP therapy in patients with comorbid mental illness, particularly when psychiatric symptoms are stable and well-managed. Clinicians should not assume reduced compliance in this population; instead, they should emphasize early and continuous patient education, regular follow-up, and integration of psychiatric care into sleep management. Delayed symptom resolution should not be interpreted as therapeutic failure but may instead reflect gradual neurobehavioral adaptation to improved sleep architecture.

Furthermore, the slightly higher PAP adherence observed among psychiatric patients suggests that structured follow-up and coordinated care can mitigate potential barriers to adherence. Integrating mental health support into PAP adherence programs may further enhance treatment outcomes.

The present study has limited applicability to patients with more severe or unstable psychiatric conditions. Individuals with acute psychiatric decompensation, recent hospitalization, severe cognitive impairment, untreated psychosis, or active substance use disorders were excluded to ensure treatment feasibility and ethical participation; consequently, the psychiatric subgroup primarily comprised patients with stable and adequately treated mental disorders under regular follow-up. The findings, therefore, cannot be generalized to patients with severe, unstable, or treatment-resistant psychiatric illness, in whom adherence patterns, executive functioning, insight, motivation, medication burden, and social stability may differ substantially. In more severe psychiatric populations, factors such as cognitive dysfunction, negative symptoms, impaired illness insight, sedation related to psychotropic medications, disrupted circadian regulation, and limited access to structured follow-up may negatively influence both PAP adherence and long-term treatment outcomes, as well as the perception and reporting of daytime sleepiness. Thus, while the present results support the feasibility and effectiveness of PAP therapy in carefully selected patients with stable psychiatric disorders, caution is warranted when extrapolating these conclusions to broader or more complex psychiatric populations, and future studies should specifically examine adherence and outcomes in individuals with more severe psychiatric presentations using structured cognitive and behavioral assessments.

## 5. Limitations

This study has several limitations that should be acknowledged. First, psychiatric diagnoses were not stratified by type or severity in the primary analyses. Although the distribution of psychiatric disorders was described, grouping heterogeneous conditions into a single category may have obscured potential differential effects between diagnostic subgroups, such as major depressive disorder, bipolar disorder, or schizophrenia spectrum disorders. Future studies with larger subgroup samples are warranted to explore diagnosis-specific adherence patterns and treatment outcomes.

Second, although psychiatric stability was required for inclusion, psychotropic medications were not analyzed as independent variables. Antidepressants, antipsychotics, mood stabilizers, and sedative–hypnotics may influence sleep architecture, daytime alertness, and potentially PAP adherence. The absence of medication-stratified analyses limits the ability to determine whether pharmacological treatment contributed to observed outcomes.

Third, while PAP adherence was objectively recorded using device-generated data, treatment effectiveness was primarily assessed using the ESS, a validated but subjective measure of daytime sleepiness. ESS scores may be influenced by mood state, perception, and reporting bias. The study did not include standardized psychiatric symptom severity scales or health-related quality-of-life instruments, limiting conclusions regarding broader psychosocial or mental health improvements.

Although the study followed participants for up to five years, the number of participants decreased substantially across follow-up visits. This reduction reflects both staggered enrollment and loss to follow-up and may introduce attrition bias. While linear mixed-effects modeling allows inclusion of incomplete cases under a missing-at-random assumption, the smaller sample size at later time points reduces statistical power and may limit the precision of long-term estimates.

An additional limitation is that validated psychiatric symptom severity scales or standardized quality-of-life questionnaires were not administered during follow-up. Although psychiatric diagnoses and treatment stability were documented, the study did not include longitudinal assessment of mood, anxiety severity, or broader psychosocial functioning. Therefore, while improvements in daytime sleepiness were observed, conclusions regarding the impact of PAP therapy on mental health status or quality of life cannot be directly inferred.

Future research should employ more granular psychiatric categorization and include additional metrics of cognitive and functional recovery beyond sleepiness. Incorporating qualitative assessments could also help elucidate motivational and perceptual barriers to adherence. Additionally, investigating the effects of psychiatric medication type and dosage on PAP efficacy and symptom resolution would be highly informative. Studies exploring integrated care models—where sleep specialists and psychiatrists collaborate in the management of patients—may yield strategies to further enhance long-term outcomes in this dual-diagnosis population.

Another limitation is the lack of standardized objective respiratory efficacy outcomes under PAP therapy, such as device-derived residual AHI, mask leak, and pressure metrics, and the absence of routine post-treatment PSG reassessment. Although such parameters can provide direct evidence of physiological control of sleep-disordered breathing, they were not consistently available across device types and time points in this real-world cohort. As a result, treatment effectiveness in the present study was inferred primarily from changes in ESS and cardiometabolic markers (BMI, blood pressure, and heart rate). This approach may be particularly vulnerable to reporting bias in psychiatric populations, where subjective sleepiness can be influenced by mood fluctuations and psychotropic medications. Future prospective studies should incorporate harmonized device-derived respiratory efficacy data and/or standardized follow-up PSG protocols, alongside validated psychiatric symptom severity and quality-of-life instruments, to better capture multidimensional treatment response.

## 6. Conclusions

This study demonstrates that patients with stable and treated mental disorders and moderate-to-severe OSA can achieve adherence to PAP therapy comparable to that of patients without psychiatric comorbidity during long-term follow-up. Contrary to the common assumption that psychiatric illness inherently compromises treatment compliance, our findings do not support a negative independent effect of psychiatric comorbidity on PAP adherence or longitudinal improvement in daytime sleepiness.

Although individuals with psychiatric diagnoses initially exhibited numerically higher symptom burden at certain follow-up points, multivariable-adjusted analyses confirmed that ESS trajectories did not significantly differ between groups. These findings, therefore, support the feasibility and clinical effectiveness of PAP therapy in carefully selected patients with stable psychiatric conditions, rather than suggesting enhanced adherence or superiority. Clinically, psychiatric comorbidity alone should not be regarded as a contraindication or barrier to initiating PAP therapy when the psychiatric condition is stable and appropriately managed.

Importantly, the present results apply specifically to patients with stable, treated mental disorders, as individuals with unstable psychiatric disease or severe cognitive impairment were excluded. Additionally, substantial attrition over the follow-up period warrants cautious interpretation, as residual selection bias cannot be entirely excluded despite baseline comparability between completers and non-completers.

Overall, our findings reinforce the importance of identifying and managing OSA in patients with stable psychiatric comorbidity and support integrated care approaches combining sleep medicine and mental health services. Future research should further explore disorder-specific effects, the impact of psychotropic medications, and strategies to optimize long-term adherence in broader psychiatric populations.

## Figures and Tables

**Table 1:** Baseline anthropometric characteristics.

Parameter	Group A (Mean ± SD)	Group B (Mean ± SD)	*p*-Value
Gender (Female)	115 (52.0%)	533 (36.7%)	0.000
Age (years)	55.08 ± 12.32	57.59 ± 13.42	0.006
BMI (kg/m^2^)	32.85 ± 6.44	32.03 ± 6.60	0.087
Waist (cm)	102.82 ± 35.94	100.57 ± 38.26	0.420
Hip (cm)	101.80 ± 34.62	100.56 ± 37.42	0.648
Neck (cm)	36.76 ± 12.43	35.60 ± 13.35	0.232
Systolic BP	102.43 ± 47.77	102.93 ± 49.15	0.886
Diastolic BP	65.58 ± 30.55	64.37 ± 30.98	0.588
Heart Rate (bpm)	81.13 ± 20.40	75.10 ± 16.13	0.013
ESS	8.14 ± 4.99	8.57 ± 5.08	0.269
FVC Predicted (%)	91.41 ± 17.57	91.48 ± 18.86	0.955
FEV_1_ Predicted (%)	90.94 ± 18.70	90.29 ± 20.12	0.645

SD, standard deviation; BMI, body mass index; BP, blood pressure; bpm, beats per minute; ESS, Epworth Sleepiness Scale; FVC, forced vital capacity; FEV_1_, forced expiratory volume in one second.

**Table 2:** Baseline sleep characteristics of the participants.

Parameter	Group A (Mean ± SD)	Group B (Mean ± SD)	*p*-Value
Total Sleep Time (min)	277.35 ± 97.83	247.71 ± 103.43	0.000
Sleep Efficiency	76.44 ± 18.23	78.95 ± 18.03	0.092
Sleep Stage 1 (%)	10.89 ± 16.56	14.20 ± 17.77	0.017
Sleep Stage 2 (%)	63.83 ± 23.05	57.26 ± 22.99	0.001
Sleep Stage 3 (%)	17.38 ± 16.27	20.39 ± 20.24	0.040
REM (%)	8.56 ± 11.05	9.57 ± 8.93	0.272
AHI	35.01 ± 30.54	38.36 ± 27.94	0.13
Mean SaO_2_	92.02 ± 3.01	92.26 ± 3.10	0.293
Lowest SaO_2_	81.82 ± 8.84	80.04 ± 9.69	0.007
ODI	36.77 ± 32.07	31.96 ± 26.97	0.199

SD, standard deviation; REM, rapid eye movement; AHI, apnea–hypopnea index; SaO_2_, arterial oxygen saturation; ODI, oxygen desaturation index.

**Table 3:** Baseline Characteristics of Participants Who Completed Long-Term Follow-Up Versus Those Lost to Follow-Up.

Parameter	Completed Follow-Up (n = 69) Mean ± SD	Lost to Follow-Up (n = 1603) Mean ± SD	*p*-Value
Age (years)	58.91 ± 12.10	57.19 ± 13.40	0.29
Female (%)	22 (31.9%)	626 (39.1%)	0.21
BMI (kg/m^2^)	31.52 ± 5.88	32.24 ± 6.61	0.34
ESS	8.34 ± 4.76	8.54 ± 5.07	0.71
AHI	36.92 ± 26.81	38.03 ± 28.74	0.73
ODI	32.88 ± 24.15	32.54 ± 28.04	0.91
Lowest SaO_2_ (%)	80.87 ± 8.72	80.34 ± 9.63	0.64
Heart Rate (bpm)	76.85 ± 18.01	75.79 ± 16.71	0.62
Psychiatric Diagnosis (%)	9 (13.0%)	212 (13.2%)	0.96

## Data Availability

The data presented in this study are available upon reasonable request from the corresponding author. The data are not publicly available due to ethical and privacy restrictions related to sensitive patient information.

## Abbreviations

The following abbreviations are used in this manuscript:

AHI	Apnea–Hypopnea Index
APAP	Auto-Adjusting Positive Airway Pressure
BMI	Body Mass Index
BP	Blood Pressure
CPAP	Continuous Positive Airway Pressure
ESS	Epworth Sleepiness Scale
FEV_1_	Forced Expiratory Volume in One Second
FVC	Forced Vital Capacity
ODI	Oxygen Desaturation Index
OSA	Obstructive Sleep Apnea
PAP	Positive Airway Pressure
PG	Polygraphy
PSG	Polysomnography
REM	Rapid Eye Movement
REML	Restricted Maximum Likelihood
SaO_2_	Arterial Oxygen Saturation
SD	Standard Deviation
